# Where Do Reproductive-Aged Women Want to Get Contraception?

**DOI:** 10.1089/jwh.2022.0406

**Published:** 2023-06-06

**Authors:** Megan L. Kavanaugh, Mia R. Zolna

**Affiliations:** Research Division, Guttmacher Institute, New York, New York, USA.

**Keywords:** contraception, reproductive health, health care access, telehealth, patient-centered care

## Abstract

**Background:**

People's preferences regarding how they want to obtain contraception should be considered when building and refining high-quality contraceptive care programs, especially in light of recent shifts to incorporate more telehealth options into contraceptive care due to the coronavirus disease 2019 (COVID-19) pandemic.

**Methods:**

Our study is a cross-sectional analysis of population-representative surveys conducted between November 2019 and August 2020 among women aged 18–44 years in Arizona (*N* = 885), New Jersey (*N* = 952), and Wisconsin (*N* = 967). We use multivariable logistic regression to identify characteristics associated with each of five contraception source preference groups (in-person via health care provider, offsite with a provider via telemedicine, offsite without a provider via telehealth, at a pharmacy, or via innovative strategies), and we examine associations between contraceptive care experiences and perceptions and each preference group.

**Results:**

Across states, most respondents (73%) expressed preferences for obtaining contraception via more than one source. One quarter indicated a narrow preference for obtaining contraception in-person from a provider, 19% expressed interest in doing so offsite with a provider via telemedicine, 64% for doing so offsite without a provider via telehealth, 71% reported interest in pharmacy-based contraception, and 25% indicated interest in getting contraception through innovative strategies. Those who had experienced nonperson-centered contraceptive counseling reported higher levels of interest in telehealth and innovative sources, and those who expressed mistrust in the contraceptive care system had higher levels of preferring to obtain contraception offsite, via telemedicine, telehealth, and other innovative avenues.

**Conclusions:**

Policies that ensure access to a diversity of contraceptive sources, which acknowledge and address people's past experiences of contraceptive care, have the greatest likelihood of closing the gap between people's contraceptive access preferences and realities.

## Introduction

Contraception is a key tool that helps people to realize reproductive autonomy in building families that align with their life circumstances and personal decisions. The most commonly used contraceptive methods in the United States typically require some sort of contact with a health care provider, whether it involves obtaining a surgical tubal ligation, a prescription for an intrauterine device (IUD).^1^ In 2015–2019, 41% of reproductive-aged women* in the United States (26 million) received a contraceptive service, most commonly from a private provider, although women who were younger, lower income, people of color, born outside of the United States, or uninsured all more commonly received contraceptive care from publicly funded health care centers than did their counterparts.^2^

In 2020, 44% of reproductive-aged women using contraception nationally indicated that their recent contraceptive care was patient-centered,^3^ meaning that their provider met their needs in the domains of interpersonal connection, adequate information, and decision support during their health care interactions.^4^ More recently in 2021, during the second year of the coronavirus disease 2019 (COVID-19) pandemic, almost 20% of reproductive-aged people assigned female at birth who received recent contraceptive care indicated having done so via telehealth; these telehealth contraceptive visits were rated as less patient-centered than in-person visits.^5^

These differences in where people obtain contraceptive care and in their experiences of this care highlight the importance of attending to issues of equity, access, and quality when developing contraceptive care programs.^6^ This is especially timely to consider, given the rapid expansion of contraceptive service delivery via more innovative systems—such as telehealth care—since the onset of the COVID-19 pandemic.^7,8^ Telehealth contraceptive care delivery holds many promises for reducing access barriers experienced via traditional medical care settings but expanding services via this mode of delivery in service of easier access must be done in a way that does not compromise quality.

Prioritizing people's preferences around both what contraceptive methods align with their life circumstances and how they want to obtain these methods when setting up or refining contraceptive care delivery programs is an essential step to ensuring that the available care meets people's needs. Recent studies have shed light on differences between contraceptive methods used and methods people prefer to use.^9–11^ Just as there is no one contraceptive method that meets everyone's needs and preferences, so too is there not one mode of method delivery that meets everyone's needs.

This study documents where and how reproductive-aged women in three select states want to get their contraceptive method across a diversity of possible options, both traditional and more innovative. In addition, we examine the relationship between people's past experiences and mistrust around accessing contraceptive care within the health care system and their preferences for five different types of contraceptive procurement: in-person, offsite via a provider through telemedicine, offsite and not involving interaction with a health care provider through telehealth, via a pharmacy, and through other innovative strategies.

These findings complement the existing literature regarding where people obtain contraception by expanding understanding of potential gaps between the available sources of contraception procurement and those that are preferred. Given the recent shifts in the abortion access landscape in the United States due to the SCOTUS Dobbs v. Jackson Women's Health Organization decision and resulting renewed attention to improve contraception access to mitigate some of the impacts of those shifts, these findings are especially timely and informative.

## Methods

### Data

Data for this analysis come from the baseline Arizona, New Jersey, and Wisconsin Surveys of Women (SoWs), longitudinal population-based surveys conducted simultaneously by the nonpartisan and objective research organization NORC at the University of Chicago between November 2019 and August 2020. SoWs were conducted in these specific states to align with a broader study, the Reproductive Health Impact Study,^12^ which examines the impact of differing policy changes on family planning care. These three states have different reproductive health landscapes in terms of access and legislative support for this access, with New Jersey representing a state with a long-standing history of enacting policies that are generally supportive of sexual and reproductive health, and both Arizona and Wisconsin being states with a mix of supportive and hostile policies in this sphere.

The SoWs are self-administered surveys focused on sexual and reproductive health experiences and attitudes and are representative of the population of reproductive-aged adult women (18–44 years) in each state. NORC randomly sampled households in each of these states using address-based sampling methods enhanced with an age-targeted list and demographic information from the American Community Survey; these sampling procedures were similar to related surveys in other states.^13–15^ NORC oversampled census tracts with higher proportions of low-income and non-White census tracts in each state. NORC initially mailed invitations to participate that included information on how to complete the questionnaire online and later mailed paper surveys to nonrespondent households, making a total of six requests for participation.

Individuals were eligible to participate if they self-identified as female, transgender, or gender expansive, were 18 to 44 years old, and resided in a sampled household.^†^ The response rate was 29% overall, specifically 32% in Arizona, 24% in New Jersey, and 38% in Wisconsin. To account for nonresponse, base sampling, adjustment for unknown eligibility and household size, and poststratification, NORC provided statistical weights to ensure that the sample represented the demographics of women aged 18 to 44 years in each of the three states. NORCs Institutional Review Board approved the data collection protocols. Given the de-identified nature of the survey data shared with the research team, this secondary data analysis was exempt from further review.

### Measures

Our analysis focuses on responses to the following contraception source item: “If you could get your birth control method from any of the following locations, or in any of the following ways, which options would you prefer?” This item included several response options, allowed for selection of multiple responses, and offered an open-ended write-in response option. Only respondents who had indicated that they had been using a method of birth control in the 3 months before completing the questionnaire were eligible to answer this contraception source item.

The exact question wording assessing current use of contraception was: “In the past 3 months, have you or a partner with whom you have had penile-vaginal sex or sex that could lead to pregnancy used any method or methods of birth control?” Of the original 6209 respondents to the surveys across the three states, 48% indicated “yes” to this item. These are the individuals who were eligible to respond to the contraception source preference items of focus in this analysis. We narrowed our analytic sample to respondents who indicated at least one contraception source preference.

We consider each contraception source preference broadly, allowing for multiple preferences, and grouped according to source (in-person provider interaction, telemedicine, telehealth, pharmacy, and other strategies). Respondents were considered as having an *in-person* provider interaction preference for obtaining contraception if they indicated either of the following response options: “at a doctor's office while there for an in-person visit” or “at a walk-in clinic that offers many reproductive health services like STI screening or free condoms.” A preference for contraception obtained offsite via *telemedicine* included those who indicated the following response option: “through telemedicine (when you speak with a healthcare provider over the computer or phone).”

Respondents were considered as having a preference for obtaining contraception outside of the formal health care system offsite without provider interaction via *telehealth* if they indicated any of the following response options: “through an app on my phone,” “ordered online but I go to a convenient place to pick it up (like Amazon pick-up),” or “ordered online such as through Amazon and shipped to my home.” Preferences for obtaining contraception via a *pharmacy* included the following response options: “at the pharmacy with a prescription from my healthcare provider,” “from a pharmacist who will write a prescription for my method,” “directly from the pharmacy without a prescription,” and “over-the-counter without a prescription (like how you buy Advil or Tylenol).”

Preferences for *other strategies* for obtaining contraception included the following response options: “delivered to my home through the mail,” “through a service that delivers to my home (like UberEats or GrubHub),” “delivered to my house by a drone (flying machine that delivers packages directly to your door),” “from a vending machine,” or “from a bicycle messenger.” Write-in responses that aligned with any of the possible contraception source preferences offered were incorporated into those preferences or the related group if broader than the individual options; in instances where a contraception source preference write-in was unclear or did not align with any of the existing preference options or groups, we did not include it (*n* = 5).

To understand associations between respondents' past experiences with, and perceptions of, contraceptive care and their desires for where to get contraception in the future, we examine two key independent variables: receipt of person-centered contraceptive counseling (PCCC) and mistrust of the contraceptive care health system. The first of these draws on the PCCC metric,^16^ which includes four items asking respondents to rate their most recent contraceptive provider (provider of any of the following types of contraceptive care in the previous 12-month period: a method of birth control or a prescription for a method, a checkup or medical test related to using a method, or information/counseling about a method) on a Likert scale: respecting the respondent as a person, letting the respondent say what mattered to them about birth control, taking the respondent's preferences about their birth control seriously, and giving the respondent enough information to make the best decision about their birth control.

Following published guidance,^17^ we created a three-category variable that considered respondents who indicated excellent on all four items as having received person-centered contraceptive care, those who had received recent contraceptive care but who did not indicate excellent on all four items as having received less than excellent contraceptive care, and all others who had not received recent contraceptive care. The one respondent who reported “prefer not to answer” to all four PCCC items was excluded from the denominator.

We created a dichotomous composite variable representing mistrust of the contraceptive care system, which draws on four items measured on a Likert scale and adapted from past surveys assessing medical mistrust:^18–20^ “the government makes certain that birth control methods are safe before they come onto the market,” “the government and public health institutions use poor people and people of color as guinea pigs to try out new birth control methods,” “the government is trying to limit populations of color by encouraging their use of birth control,” and “drug companies don't care if birth control is safe, they just want people to use it so they can make money.” Respondents who reported either disagree or strongly disagree on the first item or agree or strongly agree on any of the remaining items are considered to have mistrust of the contraceptive care system and all other respondents are not; respondents who reported “prefer not to answer” to all four items were excluded from the denominator.

Hypothesizing that characteristics associated with contraceptive use patterns would likely also be associated with preferences for where to get contraception, we examined the following respondent characteristics: state of survey, age, race and ethnicity, sexual orientation, educational attainment, employment, income as a percentage of the federal poverty level, relationship status, health insurance coverage, and use of a provider-involved contraceptive method. This last variable comprises three categories: use of a method that typically does not require interaction with a health care provider at initiation (*e.g.,* partner vasectomy, withdrawal, condoms, barrier methods, fertility awareness-based methods, emergency contraception, or another method reported via write-in), use of a method that typically requires interaction with a health care provider at initiation (*e.g.,* implant, IUD, or tubal ligation), and use of a method that typically requires more regular interaction with a health care provider (*e.g.,* oral contraceptive pill, patch, vaginal ring, and shot).

### Statistical analyses

We first conducted descriptive analyses of the sample, by state and pooled across states. Also, by state, we calculated the percentage of the population who indicated a preference for obtaining contraception via each of the possible response options provided and who fell into each of the contraception source preference groups. Given the similar patterns in preferences expressed across the three states, we pooled data across them and ran simple (not presented) and multivariable logistic regression models to examine associations between respondent characteristics and each of the five dependent variables representing the contraception source preference groups.

Given that the majority of contraceptive care has historically been provided in in-person health care settings and we sought to understand who held preferences for obtaining contraception solely via this traditional mode, we created a narrow *in-person* preference group limited to individuals who reported a preference for obtaining *in-person* contraception and simultaneously did not report a preference for obtaining contraception via *telemedicine* or *telehealth* avenues (see definitions above) for the first model. All other contraception source preference groups were examined broadly as dependent variables in the subsequent four models.

Finally, we ran additional simple and multivariable models among the pooled sample, two for each of the five dependent variables, to examine associations between the key independent variables of receipt of person-centered contraceptive care and mistrust of the contraceptive care health system and each contraceptive preference group. All respondent demographic characteristics presented were included as covariates in the multivariable models, both given their theoretical relevance to understanding contraceptive preferences due to each being associated with other contraceptive outcomes in the scientific literature and because, in our data, all bivariate associations between each characteristic and at least one of the key outcomes were significant at the *p* < 0.1 level.

The survey fielding period started before the onset of the COVID-19 pandemic and continued through its early months. To check whether the pandemic shifted preferences for contraception sources, we also conducted sensitivity analyses comparing overall preferences for contraception between two groups: “pre-COVID,” including respondents who completed the survey between November 2019 and March 10, 2020, and “during COVID,” including respondents who completed the survey between March 11, 2020—the day that the COVID-19 pandemic was declared in the United States—and August 2020. All analyses were conducted using NORC-provided weights to represent the reproductive-aged adult population of women in each state; analyses were performed within Stata version 17.0 (College Station, TX).

## Results

### Demographics

Our final analytic sample includes 885 respondents in Arizona, 952 respondents in New Jersey, and 967 respondents in Wisconsin who indicated a preference for where/how to get contraception, for a total analytic sample of 2804 (Table 1). Women of reproductive age differed somewhat in their demographic characteristics based on their state; most notably, 40% of Arizonans identified as Hispanic, while 18% of New Jerseyans and 5% of Wisconsinites did so. A majority identified as age 18–34 years, straight, having at least some college- or associate-level education, employed, higher income, married or cohabiting, having private health care insurance, and using a contraceptive method that involved at least some level of contact with a health care provider. About a third of the pooled sample (32%) reported having received recent person-centered contraceptive care, and 36% reported having some level of mistrust in the contraceptive health care system.

**Table 1.** Demographic and Contraceptive Care-Related Characteristics Among Women Aged 18–44 Years in Arizona, New Jersey, and Wisconsin, 2019–2020

	Arizona	New Jersey	Wisconsin	Pooled sample across states
	*N* = 885	*N* = 952	*N* = 967	*N* = 2804
	Weighted %	Weighted %	Weighted %	Weighted %
Total	100	100	100	100
Age, years				
18–24	31	25	27	27
25–29	22	22	23	23
30–34	19	20	17	19
35–39	15	19	18	17
40–44	12	14	15	14
Race and ethnicity				
White non-Hispanic	50	59	83	63
Black non-Hispanic	3	9	5	6
Multiracial or other non-Hispanic	6	14	7	9
Hispanic	40	18	5	22
Sexual orientation				
Straight	87	89	88	88
Lesbian, gay, bisexual, queer, pansexual, or other	11	9	11	10
Educational attainment				
HS graduate, GED, or less	16	12	13	14
Some college or associate degree	51	32	45	42
College graduate or more	33	56	42	44
Employment^a^				
Employed	73	77	81	77
Unemployed	3	4	3	3
Out of the labor market	24	19	16	20
Income as a % of the federal poverty level				
Below 100%	13	7	12	10
100%–199%	18	9	16	14
200% or higher	64	79	68	71
Relationship status				
Married	42	41	40	41
Cohabiting	26	17	26	23
Never married, not cohabiting	28	39	31	33
Formerly married, not cohabiting	4	3	2	3
Health insurance coverage^b^				
None	11	6	6	8
Private	71	79	79	76
Public	15	10	11	12
Current method use^c^				
No contact with provider	26	35	26	29
Minimal/initiation contact with provider	31	16	28	25
Regular contact with provider	42	48	46	46
Past receipt of person-centered contraceptive care^d^				
No care	39	39	42	40
Less than excellent care	31	28	23	28
Excellent care	30	32	35	32
Mistrust in the contraceptive care health system^e^				
No	62	65	62	63
Yes	38	35	37	36

State samples include respondents who reported using contraception in the 3 months before the survey and who indicated at least one preference for a source of contraception; samples are weighted to reflect women aged 18–44 years within each state. Some characteristics do not sum to 100% due to nonresponse.

^a^ Respondents who were out of work for less than a year or more were considered to be unemployed and those who were retired or a full-time student or homemaker were considered to be out of the labor market.

^b^ Private insurance includes employer-based plans and plans purchased on the marketplace or exchange. Public insurance options include Medicaid, Medicare, Tricare, Indian Health Service, and State Family Planning Program.

^c^ No contact with provider methods include withdrawal, internal and external condoms, other barrier methods, fertility awareness-based methods, emergency contraceptives and spermicides, and vasectomy. Minimal/initiation contact with provider methods include the implant, IUD, and tubal ligation. Regular contact with provider methods include the pill, patch, ring, and Depo-Provera^®^.

^d^ Respondents were considered to have received person-centered care if they reported having received a contraceptive-related care visit in the prior 12 months, and they rated this care as excellent on each of the following four domains: respecting the respondent as a person, letting the respondent say what mattered to them about birth control, taking the respondent's preferences about their birth control seriously, and giving the respondent enough information to make the best decision about their birth control; respondents who had not received contraceptive care in the past 12 months were categorized as having received no care.

^e^ Respondents were considered to have mistrust in the contraceptive health care system if they reported either disagree or strongly disagree on “the government makes certain that birth control methods are safe before they come onto the market,” or agree or strongly agree on either “the government and public health institutions use poor people and people of color as guinea pigs to try out new birth control methods,” “the government is trying to limit populations of color by encouraging their use of birth control,” or “drug companies don't care if birth control is safe, they just want people to use it so they can make money.”

IUD, intrauterine device.

### Preferred contraception sources

Respondents could report preferences for multiple sources of contraception (Table 2). Across the three states, there was broad interest (>50%) in obtaining contraception through an in-person provider interaction (doctor visit or walk-in clinic appointment), telehealth (phone app or ordering online for either pick up or a home delivery), and at a pharmacy (with or without a prescription). Less than a quarter of women in each of the states indicated a preference for obtaining contraception via telemedicine, an offsite visit with a health care provider, and around a quarter in each state indicated a preference for obtaining contraception via an innovative strategy such as nontraditional delivery options, drones, vending machines, or bike messengers. Across the three states, most women indicated a preference for obtaining contraception via multiple source groups: 27% reported a preference within only one of the five groups, 28% reported a preference in two of the five groups, and 45% reported a preference in three or more of the five groups (data not shown in tables).

**Table 2.** Preferred Source of Contraception Among Women Aged 18–44 Years in Arizona, New Jersey, and Wisconsin, 2019–2020

	Arizona	New Jersey	Wisconsin
	*N* = 885	*N* = 952	*N* = 967
	Weighted %	Weighted %	Weighted %
**Total**	100	100	100
**Preferred source of contraception** ^a^			
In-person provider interaction	**70**	**63**	**69**
Doctor visit	65	59	63
Walk-in clinic appointment	26	16	24
**Telemedicine**	**22**	**17**	**19**
Telehealth	**64**	**68**	**58**
Ordered online and pick up	21	20	18
Ordered online for delivery to home	52	60	49
Phone app	37	33	32
**Pharmacy**	**70**	**76**	**67**
Over the counter w/o Rx or store	47	46	40
Pharmacy w/Rx from doctor	41	48	44
Pharmacy w/o Rx	33	35	31
Pharmacist-provided Rx	18	15	18
**Innovative strategies**	**27**	**26**	**22**
Other nontraditional delivery service (*e.g.,* Uber)	21	19	13
Delivery by drone	14	11	8
Vending machine	13	11	11
Bike messenger	5	4	5

State samples include respondents who reported using contraception in the 3 months before the survey and who indicated at least one preference for a source of contraception; samples are weighted to reflect women aged 18–44 years within each state.

^a^ Preferences for contraception sources are not mutually exclusive; respondents could indicate as many preferences as they desired. Grouped preferences are presented in bold and reflect the percentage of respondents who indicated at least one of the preferences included within that group.

Among possible options for obtaining contraception in-person from a health care provider, a preference for doing this via a doctor visit (59%–65%) was reported more commonly than a preference for a walk-in clinic appointment (16%–26%). Among possible telehealth contraception source options, a preference for ordering contraception online for a home delivery (49%–60%) was more commonly reported than ordering online for pick up (18%–21%) or obtaining contraception via a phone app (32%–37%).

With regard to preferences for obtaining contraception via a pharmacy, using a doctor's prescription to get contraception at the pharmacy (41%–48%) and obtaining contraception over the counter without a prescription (40%–47%) were the two most commonly reported preferences in this group, while having a pharmacist directly provide a prescription for contraception was the least commonly reported (15%–18%). Finally, among other strategies for obtaining contraception, 21% or fewer indicated a preference for any of the other possible contraception source strategies offered (nontraditional delivery services such as Uber, delivery via drone, vending machine, or bike messenger).

### Characteristics and contraceptive care experiences associated with preferences for contraception sources

Overall, 24% of women of reproductive age in these three states reported a narrow preference for obtaining contraception in-person from a health care provider, 19% indicated a preference for obtaining contraception via telemedicine, 64% indicated a preference for obtaining contraception via telehealth, 71% stated a preference for obtaining contraception at a pharmacy, and 25% reported wanting to get contraception through innovative strategies (Table 3). Notably, women in New Jersey and Wisconsin reported lower levels of preferences for obtaining contraception via telemedicine than did Arizonan women (adjusted odds ratio [aOR] = 0.6 in New Jersey and 0.7 in Wisconsin, *p* ≤ 0.02), and Wisconsin women also reported lower levels of preferences for pharmacy sources for contraception than did women in Arizona (aOR = 0.7, *p* = 0.04).

**Table 3.** Associations Between Demographic Characteristics and Contraception Source Preference Groups Among Women Aged 18–44 Years in Arizona, New Jersey, and Wisconsin (Pooled *N* = 2804), 2019–2020

	Narrow preference for obtaining contraception in-person from a health care provider^a^		Preference for obtaining contraception via telemedicine^b^		Preference for obtaining contraception via telehealth^c^		Preferences for obtaining contraception via a pharmacy^d^		Preferences for obtaining contraception via innovative strategies^e^	
	Weighted %	aOR (95% CI)	Weighted %	aOR (95% CI)	Weighted %	aOR (95% CI)	Weighted %	aOR (95% CI)	Weighted%	aOR (95% CI)
Overall	24%		19%		64%		71%		25%	
State										
Arizona	34%	Ref.	38%	Ref.	33%	Ref.	33%	Ref.	36%	Ref.
New Jersey	32%	1.09 (0.74–1.61)	33%	0.57 (0.40–0.81)	40%	1.02 (0.74–1.40)	40%	1.02 (0.73–1.45)	39%	0.96 (0.69–1.35)
Wisconsin	34%	1.32 (0.94–1.86)	29%	0.66 (0.47–0.93)	27%	0.78 (0.58–1.05)	27%	0.72 (0.52–0.99)	25%	0.78 (0.55–1.10)
Demographic characteristics										
Age, years										
18–24	27%	Ref.	21%	Ref.	28%	Ref.	29%	Ref.	35%	Ref.
25–29	22%	1.11 (0.69–1.77)	26%	1.47 (0.92–2.37)	23%	0.96 (0.64–1.42)	23%	0.77 (0.48–1.25)	22%	0.74 (0.49–1.11)
30–34	18%	1.26 (0.75–2.12)	22%	1.53 (0.95–2.47)	19%	0.87 (0.56–1.35)	19%	0.67 (0.41–1.11)	21%	1.06 (0.70–1.61)
35–39	17%	1.11 (0.68–1.83)	19%	1.47 (0.90–2.42)	18%	0.9 (0.58–1.39)	16%	0.71 (0.43–1.19)	15%	0.7 (0.46–1.09)
40–44	17%	1.56 (0.93–2.60)	12%	0.94 (0.57–1.57)	13%	0.81 (0.52–1.26)	13%	0.57 (0.34–0.94)	8%	0.5 (0.31–0.80)
Race and ethnicity										
White non-Hispanic	62%	Ref.	66%	Ref.	62%	Ref.	65%	Ref.	55%	Ref.
Black non-Hispanic	7%	1.1 (0.60–2.00)	7%	1.2 (0.64–2.24)	5%	0.73 (0.41–1.30)	5%	0.66 (0.38–1.15)	7%	1.4 (0.78–2.49)
Multiracial or other non-Hispanic	8%	0.96 (0.54–1.69)	9%	0.95 (0.57–1.58)	10%	1.2 (0.75–1.92)	10%	0.87 (0.49–1.54)	13%	1.8 (1.12–2.87)
Hispanic	23%	1.08 (0.72–1.64)	18%	0.79 (0.52–1.21)	22%	1.03 (0.72–1.47)	21%	0.87 (0.60–1.26)	25%	1.28 (0.87–1.88)
Sexual orientation										
Straight	92%	Ref.	89%	Ref.	88%	Ref.	89%	Ref.	84%	Ref.
Queer, pansexual, or other	8%	0.51 (0.31–0.85)	11%	1.15 (0.71–1.86)	12%	1.87 (1.22–2.86)	11%	1.61 (0.95–2.75)	16%	2.1 (1.39–3.16)
Educational attainment										
HS graduate, GED, or less	23%	Ref.	8%	Ref.	11%	Ref.	11%	Ref.	11%	Ref.
Some college or associate degree	43%	0.5 (0.31–0.81)	41%	1.68 (0.92–3.08)	41%	1.61 (1.05–2.48)	43%	1.89 (1.19–2.99)	44%	1.73 (1.01–2.98)
College graduate or more	34%	0.36 (0.22–0.59)	51%	1.89 (1.04–3.44)	48%	2.25 (1.45–3.48)	46%	1.97 (1.23–3.14)	46%	2.13 (1.23–3.69)
Employment^f^										
Employed	75%	Ref.	78%	Ref.	78%	Ref.	76%	Ref.	74%	Ref.
Unemployed	4%	1.03 (0.45–2.36)	2%	0.77 (0.30–1.99)	3%	0.58 (0.27–1.21)	3%	0.94 (0.44–1.99)	2%	0.48 (0.22–1.05)
Out of the labor market	21%	1.07 (0.70–1.63)	20%	1.18 (0.80–1.73)	20%	1 (0.71–1.41)	21%	1.25 (0.85–1.84)	24%	1.37 (0.96–1.95)
Income as a % of the federal poverty level										
Below 100%	14%	Ref.	8%	Ref.	10%	Ref.	10%	Ref.	12%	Ref.
100–199%	19%	0.82 (0.49–1.36)	11%	0.83 (0.45–1.53)	13%	1.23 (0.77–1.97)	13%	0.81 (0.49–1.36)	12%	0.72 (0.42–1.25)
200% or higher	67%	0.74 (0.45–1.20)	81%	1.31 (0.73–2.38)	77%	1.34 (0.86–2.09)	77%	0.96 (0.58–1.57)	76%	0.91 (0.56–1.49)
Relationship status										
Married	39%	Ref.	42%	Ref.	41%	Ref.	40%	Ref.	33%	Ref.
Cohabiting	25%	0.96 (0.65–1.41)	21%	0.9 (0.62–1.30)	21%	0.95 (0.68–1.33)	22%	0.93 (0.65–1.34)	23%	1.28 (0.88–1.85)
Never married, not cohabiting	34%	1.04 (0.71–1.53)	32%	1.07 (0.75–1.52)	34%	1.12 (0.80–1.57)	35%	1.25 (0.86–1.81)	41%	1.58 (1.11–2.23)
Formerly married, not cohabiting	3%	0.67 (0.30–1.48)	5%	1.89 (0.98–3.64)	3%	1.49 (0.81–2.75)	3%	1.21 (0.63–2.33)	3%	1.59 (0.78–3.25)
Health insurance coverage^g^										
None	7%	Ref.	5%	Ref.	9%	Ref.	7%	Ref.	9%	Ref.
Private	76%	1.36 (0.79–2.35)	83%	1.48 (0.72–3.04)	80%	0.59 (0.36–0.97)	82%	2.28 (1.39–3.73)	79%	0.85 (0.50–1.42)
Public	17%	1.54 (0.83–2.86)	12%	1.59 (0.71–3.56)	11%	0.63 (0.36–1.13)	11%	1.81 (1.00–3.27)	12%	0.74 (0.40–1.37)
Current method use^h^										
No contact with provider	16%	Ref.	28%	Ref.	35%	Ref.	32%	Ref.	30%	Ref.
Minimal/initiation contact with provider	52%	6.38 (4.43–9.19)	25%	1.1 (0.75–1.59)	18%	0.3 (0.22–0.42)	15%	0.21 (0.15–0.30)	22%	0.86 (0.59–1.26)
Regular contact with provider	32%	1.21 (0.84–1.74)	47%	1.14 (0.82–1.58)	47%	0.66 (0.49–0.89)	53%	1.35 (0.98–1.86)	48%	0.96 (0.70–1.33)

State samples include respondents who reported using contraception in the 3 months before the survey and who indicated at least one preference for a source of contraception; samples are weighted to reflect women aged 18–44 years within each state. aORs come from multivariable logistic regression models including all demographic characteristics listed.

^a^ Preference expressed for obtaining contraception through either a doctor visit or walk-in clinic appointment and no concurrent preference expressed for either telemedicine or telehealth (phone app or ordering online for home delivery).

^b^ Preference expressed for obtaining contraception via telemedicine. Any other preferences may be concurrently reported.

^c^ Preference expressed for obtaining contraception via either a phone app or through online ordering for a home delivery. Any other preferences may be concurrently reported.

^d^ Preference expressed for obtaining contraception via a pharmacy with a prescription from doctor or pharmacist, over the counter without a prescription or from a store. Any other preferences may be concurrently reported.

^e^ Preference expressed for obtaining contraception via innovative strategies include nontraditional delivery service such as Uber, drone or bike messenger, or from a vending machine. Any other preferences may be concurrently reported.

^f^ Respondents who were out of work for less than a year or more were considered to be unemployed and those who were retired or a -ime student or homemaker were considered to be out of the labor market.

^g^ Private insurance includes employer-based plans and plans purchased on the marketplace or exchange. Public insurance options include Medicaid, Medicare, Tricare, Indian Health Service, and State Family Planning Program.

^h^ No contact with a provider method includes any non-provider-involved method such as withdrawal, internal and external condoms, other barrier methods, fertility awareness-based methods, emergency contraceptives and spermicides, and vasectomy. Minimal/initiation contact with a provider method includes the implant, IUD, and tubal ligation. Regular contact with a provider method includes the pill, patch, ring, and Depo-Provera^®^.

aOR, adjusted odds ratios; CI, confidence interval.

Women aged 30–34 years had marginally higher odds of reporting a preference for telemedicine contraception (aOR = 1.5, *p* = 0.08), and those aged 40–44 years had marginally higher odds of a preference for in-person contraception (aOR = 1.6, *p* = 0.09), and significantly lower odds of a preference for pharmacy (aOR = 0.6, *p* = 0.03) or innovative strategies (aOR = 0.5, *p* < 0.01) as sources for contraception compared with those aged 18–24 years. Women who identified as multiracial or another non-Hispanic racial group reported higher levels of a preference for innovative contraception sources compared with non-Hispanic white women (aOR = 1.8, *p* = 0.01).

Those who identified as lesbian, gay, bisexual, queer, pansexual, or other reported lower odds of preferences for in-person contraception (aOR = 0.6, *p* < 0.01) and marginally or significantly higher odds of preferences for telehealth contraception (aOR = 1.9, *p* < 0.01), pharmacy sources (*p* = 1.6, *p* = 0.08), and innovative contraception sources (aOR = 2.1, *p* < 0.001) than did those who identified as straight. Compared with women with a high school degree or less, those with higher levels of education reported significantly lower levels of preferences for obtaining contraception in-person from a health care provider (aORs = 0.4–0.5, *p* < 0.01) and higher levels of preferences for obtaining contraception via telemedicine, telehealth, pharmacy, and innovative sources (aORs = 1.6–2.3, *p* ≤ 0.09).

Compared with employed women, unemployed women reported marginally lower levels of interest in innovative sources for contraception (aOR = 0.5, *p* = 0.06), whereas women out of the labor market reported marginally higher levels of interest in these sources (aOR = 1.4, *p* = 0.08). Compared with married women, those who had been formerly married and were not cohabiting reported higher levels of preferences for telemedicine contraception (aOR = 1.9, *p* = 0.06) and those who had never been married and were not cohabiting reported higher levels of interest in innovative contraception sources (aOR = 1.6, *p* = 0.01). Compared with those with no health insurance, women with private health care insurance coverage reported significantly lower levels of preferences for obtaining contraception via telehealth strategies (aOR = 0.6, *p* = 0.03) and higher levels of interest in pharmacy-based sources (aOR = 2.3, *p* < 0.01); women with public insurance also indicated higher levels of interest in pharmacy-based contraception than those with no insurance (aOR = 1.8, *p* = 0.05).

Users of contraceptive methods that required only minimal contact with a health care provider had significantly higher odds of reporting preferences for obtaining contraception in-person from a health care provider (aOR = 6.4, *p* < 0.001) and significantly lower odds of reporting preferences for obtaining contraception via telehealth (aOR = 0.3, *p* < 0.001) or pharmacy (aOR = 0.2, *p* < 0.001) sources than users of contraceptive methods involving no provider contact; users of methods requiring more regular contact with a health care provider reported lower levels of interest telehealth sources (aOR = 0.7, *p* < 0.01) and marginally higher levels of interest in getting contraception at a pharmacy (aOR = 1.4, *p* = 0.07). Women's income level was not associated with any of the five contraception source preference groups.

Table 4 presents associations between women's past experiences with contraceptive care and mistrust of the contraceptive health care system and each of the distinct preference groups for sources of contraception. Controlling for respondent demographics, those who reported having received non-person-centered contraceptive care (less than excellent ratings) reported higher odds of a preference for both telehealth contraception (aOR = 1.5, *p* = 0.01) and innovative strategies for receiving contraception (aOR = 1.4, *p* = 0.09) compared with those who did receive person-centered contraceptive care. Those indicating some level of mistrust with the contraceptive health care system reported significantly higher odds of preferring to obtain contraception offsite, via telemedicine (aOR = 1.4, *p* = 0.03), via offsite telehealth strategies (aOR = 1. 4, *p* = 0.02), and via innovative strategies (aOR = 1.6, *p* < 0.001) than did those who did not report this mistrust.

**Table 4.** Associations Between Contraceptive Care Experiences and Contraception Source Preference Groups Among Women Aged 18–44 Years in Arizona, New Jersey, and Wisconsin (Pooled *N* = 2804), 2019–2020

	Narrow preference for obtaining contraception in-person from a health care provider^a^			Preference for obtaining contraception via telemedicine^b^			Preference for obtaining contraception via telehealth^c^			Preferences for obtaining contraception via a pharmacy^d^			Preferences for obtaining contraception via innovative strategies^e^		
	Weighted %	OR (95% CI)	aOR (95% CI)	Weighted %	OR (95% CI)	aOR (95% CI)	Weighted %	OR (95% CI)	aOR (95% CI)	Weighted %	OR (95% CI)	aOR (95% CI)	Weighted %	OR (95% CI)	aOR (95% CI)
Past receipt of person-centered contraceptive care^f^															
No care	36%	0.72 (0.54–0.95)	0.79 (0.54–1.16)	38%	0.91 (0.68–1.23)	0.99 (0.65–1.52)	42%	1.42 (1.10–1.84)	1.06 (0.75–1.51)	39%	0.9 (0.69–1.18)	0.92 (0.65–1.28)	36%	0.94 (0.70–1.25)	0.87 (0.59–1.28)
Less than excellent	27%	0.8 (0.58–1.12)	0.83 (0.55–1.25)	29%	1.06 (0.76–1.48)	1.23 (0.86–1.76)	29%	1.42 (1.06–1.90)	1.5 (1.09–2.07)	28%	1.07 (0.78–1.47)	1.1 (0.75–1.62)	33%	1.38 (1.01–1.90)	1.35 (0.96–1.91)
Excellent care	37%	Ref.	Ref.	33%	Ref.	Ref.	30%	Ref.	Ref.	33%	Ref.	Ref.	31%	Ref.	Ref.
Mistrust in contraceptive care health system^g^															
No	67%	Ref.	Ref.	57%	Ref.	Ref.	60%	Ref.	Ref.	62%	Ref.	Ref.	53%	Ref.	Ref.
Yes	33%	0.81 (0.62–1.06)	0.83 (0.61–1.13)	43%	1.43 (1.10–1.85)	1.36 (1.03–1.81)	40%	1.44 (1.14–1.82)	1.37 (1.06–1.76)	38%	1.19 (0.93–1.51)	1.17 (0.88–1.55)	47%	1.84 (1.43–2.37)	1.64 (1.25–2.15)

State samples include respondents who reported using contraception in the 3 months before the survey and who indicated at least one preference for a source of contraception; samples are weighted to reflect women aged 18–44 years within each state. ORs (odds ratios) come from simple logistic regression models examining associations between each of the two independent variables represent contraceptive care experiences and each of the five dependent variables representing contraception source preference groups. aORs come from multivariable logistic regression models run separately for each of the independent variables representing contraceptive care experiences and controlling for the following demographic characteristics: state, age, race and ethnicity, sexual orientation, education, employment, income, relationship status, health insurance, and provider-involved contraceptive method use.

^a^ Preference expressed for obtaining contraception through either a doctor visit or walk-in clinic appointment and no concurrent preference expressed for either telemedicine or telehealth (phone app or ordering online for home delivery).

^b^ Preference expressed for obtaining contraception via telemedicine. Any other preferences may be concurrently reported.

^c^ Preference expressed for obtaining contraception via either a phone app or through online ordering for a home delivery. Any other preferences may be concurrently reported.

^d^ Preference expressed for obtaining contraception via a pharmacy with a prescription from doctor or pharmacist, over the counter without a prescription or from a store. Any other preferences may be concurrently reported.

^e^ Preference expressed for obtaining contraception via innovative strategies include nontraditional delivery service such as Uber, drone or bike messenger, or from a vending machine. Any other preferences may be concurrently reported.

^f^ Respondents were considered to have received person-centered care if they reported having received a contraceptive-related care visit in the prior 12 months, and they rated this care as excellent on each of the following four domains: respecting the respondent as a person, letting the respondent say what mattered to them about birth control, taking the respondent's preferences about their birth control seriously, and giving the respondent enough information to make the best decision about their birth control; respondents who had not received contraceptive care in the past 12 months were categorized as having received no care.

^g^ Respondents were considered to have mistrust of the contraceptive health care system if they reported either disagree or strongly disagree on “the government makes certain that birth control methods are safe before they come onto the market,” or agree or strongly agree on either “the government and public health institutions use poor people and people of color as guinea pigs to try out new birth control methods,” “the government is trying to limit populations of color by encouraging their use of birth control,” or “drug companies don't care if birth control is safe, they just want people to use it so they can make money.”

Results from sensitivity testing among the analytic sample comparing contraception source preferences between a pre-COVID group (*N* = 1902) and a during-COVID group (*N* = 902) indicate slight shifts between these two time periods, including a five percentage point increase in preference for offsite provider interaction via telemedicine, an eight percentage point decrease in preference for telehealth sources, a four percentage point decrease in preference for pharmacy sources, and a three percentage point decrease in preference for innovative strategies (data not shown in tables). Preferences for an in-person provider interaction did not change between the two time periods.

## Discussion

Across Arizona, New Jersey, and Wisconsin, despite different state contexts with regard to legislative support for, and access to, sexual and reproductive health care, women of reproductive age report similar levels of preferences for a wide range of sources through which they would prefer to obtain contraception. These similarities in findings across differing state contexts lend some support for extrapolating people's preferences for contraception sources to other state settings where programs and policies are incorporating person-centered approaches to contraceptive access initiatives.

Some of these highly preferred sources, including interacting with a health care provider in-person or picking up from a pharmacy or store setting, are familiar strategies for accessing contraception; other popular options—such as telehealth contraception—represent more novel approaches, especially in the changing landscape of contraceptive delivery since the onset of the COVID-19 pandemic. Obtaining contraception via telemedicine was the least popular preference grouping across states, at least in the pre-to-early pandemic time period during which this study was conducted. Women's contraception source preferences should be considered against their actual sources of contraception; in 2020, three fourths of women nationally got contraception from a doctor's office and only 5% used telemedicine to do so.^3^

Our findings highlight the importance of ensuring a diversity of options through which people can access contraception and ensuring that coverage for contraception is not limited to only certain options; prioritizing one delivery mode over others would be moving away from person-centered approaches to contraceptive access. Initiatives such as access through a pharmacist^21^ and making some birth control methods available over the counter^22^ help to shift the contraceptive delivery landscape toward better meeting people's stated preferences for a diversity of options, a key aspect of sexual and reproductive health equity (SRHE) that should be the cornerstone of efforts focused on contraceptive access and care.^23^

SRHE, as defined by the Coalition to Expand Contraceptive Access (CECA), means that systems ensure that all people, across the range of age, gender, race, and other intersectional identities, have what they need to attain their highest level of sexual and reproductive health, and includes self-determining and achieving their reproductive goals.^24^ Higher levels of interest in obtaining contraception outside of the traditional onsite provider model among more educated individuals in our study are in contrast to a recent national study in which there were no differences in receiving telehealth or telemedicine contraception by education level;^5^ those with higher levels of education may have more informational exposure to the existence of a diversity of contraceptive source options, resulting in these higher levels of interest.

We also found that people who identified as being a sexual minority preferred to obtain contraception outside of a health care provider interaction; this finding aligns with other evidence that people who identify as LGBTQ+ have reported higher levels of negative health care provider interactions in the context of sexual and reproductive health care.^25^ We found few differences in contraception source preferences by income level or race/ethnicity, findings that diverge from national research indicating that reproductive-aged individuals assigned female at birth who were low income or identified as a person of color reported higher levels of receiving telehealth and telemedicine contraception than their higher income or non-Hispanic counterparts, respectively.^5^

Our findings highlighting how actual experiences and perceptions of contraceptive care are linked to preferences support prioritizing these more person-centered and experiential metrics over demographic and socially constructed ones. Policies grounded in SRHE, which prioritize those who have historically experienced the most impediments to accessing desired contraception, have the greatest likelihood of closing the gap between how people want to obtain contraception and how they actually get it.

Most people indicated several preferences for how to obtain contraception. The extent to which people are able to realize their preferences, however, is constrained by the reality of the avenues by which contraception is available to them. Not all states allow pharmacists to directly prescribe contraception,^26^ vending machines are almost exclusively the purview of emergency contraception access on select college campuses,^27^ and delivery of contraception via drones is not yet a reality. Having insurance can play a role in contraceptive access,^28^ and having no health insurance coverage is associated with higher levels of telehealth contraceptive care;^5^ less interest in telehealth contraception among insured individuals in our study may be due to the high rate of insurance coverage among the sample and/or a recognition that insurance coverage does not always extend to this mode of contraceptive delivery.

Past research has documented the link between person-centered contraceptive care and contraceptive method preferences;^9,10^ our findings extend this link to contraception source preferences. Our study highlights how past experiences of contraceptive care, especially quality ones, can play a role in future preferences for obtaining contraception; people who had received nonpatient-centered contraceptive care preferred strategies that had less contact with health care providers, such as obtaining contraception via telehealth or other innovative strategies.

While expanding contraceptive delivery via telehealth is an important supplement to existing access strategies, policies focused on this goal should attend to the importance of maintaining high-quality care in this context and should not prioritize telehealth to the exclusion of care delivery involving interactions with health care providers. Notably, a recent national study documented the lower levels of patient-centered care reported among individuals using telehealth for contraceptive care (including videoconferencing or telephone access to health care providers as well as online contraception websites and apps) compared with those who had received contraceptive care in-person;^29^ closing this gap in quality across modalities of contraceptive delivery is integral to the delivery of contraception programs grounded in SRHE.

Mistrust of the contraceptive health care system has also been linked to lower levels of contraceptive use;^20^ our findings highlight how individuals expressing these perceptions of the health care system would prefer to minimize contact with it through use of telemedicine and telehealth contraception as well as other innovative strategies that do not involve a provider. A long history of reproductive injustices perpetuated within the health care system and through provider bias related to contraception against Black, Indigenous, and people of color specifically provides context for this mistrust.^28,30,31^ Adopting a person-centered contraceptive care framework grounded in equity^6^ is one step toward acknowledging the validity of existing mistrust and working to address it in service of patients' reproductive autonomy.

The timing of data collection for this study represents both a strength and limitation; covering the period immediately before, and in the beginning few months of, the COVID-19 pandemic offers insights into how people's preferences for contraception tracked against the rapidly changing landscape at the time. Simultaneously, given this changing landscape, the extent to which preferences documented in this study can be generalized to current preferences is unclear; future research should explore the pre-COVID to early-COVID small shifts in preferences that emerged in sensitivity testing. Telehealth contraceptive care has become more common since the time of this study, and preferences may have evolved with more exposure to this option.

Given the early indications from our sensitivity analysis that preferences shifted somewhat between the pre-COVID time period and the beginnings of the during-COVID time, future research should examine the extent to which preferences continue to change over time, in tandem with changing life circumstances, and with increasing exposure to more delivery options. Although a strength of this study is the generalizability of the findings to reproductive-aged women across Arizona, New Jersey, and Wisconsin, the low survey response rates may mask differential preferences for contraception sources held by survey nonresponders.

Given the skip patterns present in the survey, our analysis only includes individuals who reported using contraception in the 3 months before completing the survey; individuals with more fluctuating contraceptive use or nonuse may have preferences for sources of contraception that are distinct from recent contraceptive users. Finally, the survey item assessing preferences for where to obtain contraception included multiple overlapping options that did not distinguish between preferences for sources of contraception initiation versus sources of contraceptive maintenance following initiation. Respondents' own use of contraception, when they initiated their method, and their interpretation of the survey item may each have influenced their reported contraception source preferences, and our analysis is unable to detect the extent of this influence among the sample.

## Conclusions

Given the national landscape with regard to increasing restrictions on abortion access in the United States, ensuring that people have access to their preferred contraceptive strategies obtained via their preferred sources is critical. This access to contraceptive care should be supplemental to, rather than a substitution for, access to abortion care. Policies to improve access to contraception should be grounded in SRHE and address the following key components, among others: accurate and comprehensive information about all contraceptive options, support to choose preferred contraception, and access to these preferences through desired avenues. Our findings are an important contribution to understanding this last component, with particular relevance to informing initiatives in ways that recognize people's past experiences of contraceptive care and value their preferences going forward.

## Notes

^*^
In this article, we use the word “women” to reflect terminology used in cited studies. However, data collection processes do not always accurately or comprehensively capture respondents' gender, and contraceptive users' gender identities are diverse; we therefore use the word “people” when generally describing individuals who can become pregnant and who seek and use contraception.

^†^
Of the 2804 individuals in the final analytic sample, 7 (<0.5%) identified as transgender or gender expansive. Given the homogeneity of our sample on gender identity, we do not include this variable in our analysis and we use the term “women” to reflect how respondents in our study overwhelmingly identified. However, people of all gender identities use contraception, and our findings are meant to be broadly generalizable to individuals using contraception across these states.
